# First Record of Mermithidae (Enoplea: Mermithida) Parasitizing *Philaenus spumarius* (Hemiptera: Aphrophoridae) in Central Italy

**DOI:** 10.2478/jofnem-2025-0019

**Published:** 2025-06-04

**Authors:** Anita Nencioni, Gaia Bigiotti, Elisabetta Gargani, Patrizia Sacchetti, Agostino Strangi, Ilaria Cutino

**Affiliations:** Council for Agricultural Research and Economics, Research Centre for Plant Protection and Certification (CREA-DC), Via di Lanciola 12/A, 50125 Florence, Italy.; Department of Agriculture, Food, Environment and Forestry (DAGRI), University of Florence, Piazzale delle Cascine 28, 50144 Florence, Italy.

**Keywords:** Aphrophoridae, natural enemy, *Philaenus spumarius*, spittlebug, terrestrial Mermithidae, vector, *Xylella fastidiosa*

## Abstract

*Philaenus spumarius* specimens were found naturally parasitized by Mermithidae nematodes in Central Italy. Nematodes infected all spittlebug instars, showing a highly variable parasitization rate according to sampling locations. Molecular analysis highlighted the presence of two distinct species that are apparently not cohabitant in the same site. However, complete taxonomic identification will occur when adult nematodes are obtained. Further research is needed to identify these two putative nematode species and to understand their ecological requirements. Furthermore, new studies aimed to elucidate the relationship between *P. spumarius* and Mermithidae nematodes, as well as the role of these parasites in regulating spittlebug populations, can be useful in identifying potential natural enemies for pest control. To our knowledge, this is the first report of Mermithidae infecting *P. spumarius* in Europe.

Mermithidae are long, slender nematodes that are obligately parasitic in invertebrates. They have been reported to be infectious to many insect taxa and other invertebrates, including spiders, crustaceans, and leeches (Poinar, 2010; Stock, 2024). Their full development goes through six stages from egg to adult (Poinar and Otieno, 1974; Poinar, 2010; Kaiser, 2020). The infective stage, named pre-parasitic juvenile, is generally active in spring when the increase in soil temperature and the availability of soil water elicit its hatching from the egg. This juvenile finds its host wandering in the soil or climbing on low-growing plants (Nickle, 1981; Kaiser, 2020). Once inside the host, mermithids develop by absorbing nutrients directly from the host’s hemolymph. After two molts, juvenile nematodes reach the post-parasitic juvenile stage and leave the host. During the emergence from the host’s body, the mermithid perforates the insect’s cuticle, causing the death of the parasitized organism. Mermithid development is completed in soil, where nematodes will become mature adults, ready to mate and reproduce (Kaiser, 2020).

Although Mermithidae have been reported on several hemipteran species, there are only a few records regarding the parasitization of Auchenorrhyncha. In Asia, the planthoppers *Nilaparvata lugens* (Stål, 1854) and *Sogatella furcifera* (Horváth, 1899) (Delphacidae) were found to be hosts of *Agamermis unka* Kaburaki & Jamamura, 1932 (Choo et al., 1989). In the United States, Nickle (1981) observed *Agamermis decaudata* Cobb, Steiner & Christie, 1923 on the leafhopper *Aceratagallia agricola* (Hamilton, 1998) (Cicadellidae), while Sperka and Freytag (1975) reported the parasitization of thirty-seven species of Auchenorrhyncha by several unknown mermithid species. Recently, Rusconi et al. (2020) observed mermithid nematodes parasitizing both nymphs and adults of *Hortensia similis* (Walker, 1851) (Cicadellidae). Regarding species belonging to the superfamily Cercopoidea, the froghopper *Aneolamia varia* (Fabricius, 1787) was found to be heavily parasitized by *Hexamermis dactylocerus* Poinar & Linares, 1985 in sugarcane fields in Venezuela (Poinar and Linares, 1985). In Ohio, Weaver and King (1954) observed the spittlebug *Philaenus spumarius* (L.) carrying *A. decaudata* specimens. A few reports have been made from Europe, where mermithids have been only occasionally reported on *Aphrophora salicina* (Goeze, 1778) (Aphrophoridae), *Macustus nigrescens* (Zetterstedt, 1828) (Cicadellidae), and *Javesella dubia* (Kirschbaum, 1868) (Delphacidae) (Weber, 1930; Helden, 2008).

European Aphrophoridae, particularly *Philaenus spumarius,* have received increasing attention in the last decades. This spittlebug is the main vector of the bacterial plant pathogen *Xylella fastidiosa* (Wells et al., 1987) subsp. *pauca* ST53 (Saponari et al., 2014; Cavalieri et al., 2019), detected in Italy in 2013 and recognized as the causal agent of the Olive Quick Decline Syndrome (OQDS) (Saponari et al., 2013; Saponari et al., 2017). More recently, *P. spumarius* was also assessed as a vector of several strains belonging to *X. fastidiosa* ssp. *multiplex* detected in Southern Europe (Cruaud et al., 2018; Nencioni et al., 2024).

Currently, this bacterium’s management is mainly based on controlling spittlebug vectors through agronomical and chemical interventions. Considering the absence of efficient biological control strategies for *P. spumarius*, improving knowledge of its natural enemies appears to be crucial to identifying potential biological control agents.

This work reports a case of parasitization of *P. spumarius* by two mermithid species in Central Italy. This study was motivated by some previous observations of Mermithidae in *P. spumarius* specimens and aimed at identifying nematode parasites of this spittlebugs and assessing the prevalence of such nematodes in *P. spumarius* populations.

## Materials and Methods

### Insect sampling and nematode collection

During spring 2024, a field sampling was carried out in eight sites (urban green areas and organic olive groves) located in Tuscany (Central Italy). In each sampling location, an area of about 1000 m^2^ was selected for the experiment. To maximize the probability of detecting mermithid parasitization in *P. spumarius*, sampling sites were chosen based on a) the occurrence of a locally abundant population of *P. spumarius;* b) the presence of undisturbed herbaceous ground cover; c) the absence of agronomic management or, at most, exclusive application of organic farming practices. Each sampling location was visited four times between April and June. Initially, 200 *P. spumarius* froths, namely the foamy envelope in which preimaginal stages develop, were collected in each sampling site. The abdomen of all *P. spumarius* juveniles occurring within these froths was dissected under a stereomicroscope, searching for nematodes. After the emergence of spittlebug adults, at least 200 *P. spumarius* specimens were collected in each sampling location using a sweep net and dissected as previously described. At least ten nematodes of each site taken from both nymphs’ and adults’ abdomens were individually stored in 1.5 ml tubes filled with 180 μl ATL Buffer supplied with QIAmp DNA Extraction Kit (Qiagen) for molecular identification, keeping them separate according to their site of collection. Moreover, five *P. spumarius* adults that showed a swollen abdomen (a sign of putative Mermithidae parasitization) were selected for each sampling location and individually placed in Petri dishes containing sterilized damp soil (in the oven at 105°C for 24h). This procedure was conducted to allow the spontaneous emergence of nematodes from spittlebugs and let them reach the adult stage in the soil. Petri dishes were maintained at room temperature (25°C) for 45 days and periodically watered to ensure a good level of moisture for nematodes.

### Molecular analyses

Nematodes collected for molecular analysis were homogenized in a 1.5 ml microcentrifuge tube using a hand pestle after 20 μl of Proteinase K 20 mg/ml was added. Samples were incubated at 55°C for 3 hours. The following DNA extraction steps were performed according to QIAmp DNA Extraction Kit (Qiagen) specifications, and the final elution step was performed in 50 μl of AE Buffer supplied with the kit. The concentration of eluted DNA was measured using a Qubit photometer (ThermoFisher).

Amplification of the ITS locus was performed in a total volume of 50 μl made with HotStart DreamTaq Master Mix 1X (ThermoFisher), 0.6 μm of each primer, and 5.0 μl of Template DNA. The thermal program was 94°C for 3 min followed by 45 cycles of denaturation at 94°C for 30 s, primer annealing at T_a_ (see Table 1) for 30 s, extension at 72°C for 60 s, and a final elongation step of 10 min at 72°C. Amplicons were checked on TapeStation 4200 (Agilent). Nematodes were subdivided according to the length of the ITS locus, and one nematode of each class was further characterized through sequencing of the ITS locus. The amplicons were purified with QIAquick PCR purification kit (QIAGEN) and sequenced in-house using SeqStudio (ThermoFisher).

**Table 1:** Primer sequences and their annealing temperatures.

**Primer name**	**Primer sequence**	**T_a_**	**Reference**
988F	CTCAAAGATTAAGCCATGC	45.0 °C	Holterman et al., 2006
26R	CATTCTTGGCAAATGCTTTCG		Nguyen and Hunt, 2007
MermF	CAAGGACGAAAGTTAGAGGTTC	47.0 °C	Kobylinski et al., 2012
MermR	GGAAACCTTGTTACGACTTTTA		
Nem1	GCAAGTCTGGTGCCAGCAGC	45.0 °C	Foucher and Wilson, 2002
Nem2	CCGTGTTGAGTCAAATTAAG		
18S	TTGATTACGTCCCTGCCCTTT	50.0 °C	Vrain et al., 1992
26S	TTTCACTCGCCGTTACTAAGG		
D2A	ACAAGTACCGTGAGGGAAAGTTG	52.0 °C	Douda et al., 2010
D3B	TCGGAAGGAACCAGCTACTA		

Other loci were considered to further characterize the same samples. The 18S gene and D2-D3 expansion region were amplified following the aforementioned protocol using primer pairs described in Table 1. Amplicon sequences were assembled, and the resulting loci were matched with the GenBank database using the BLAST algorithm to identify the most probable species.

### Statistical analysis

The Chi-square (χ^2^) Test for Independence was used to compare the parasitization rate measured in each sampling site. The significance level was set at *p*<0.05. Statistical analyses were performed using PAST Version 4.17 (Hammer et al. 2001).

## Results

Mermithid nematodes were found parasitizing both *P. spumarius* nymphs and adults in five out of eight inspected locations (Table 2). Unfortunately, the rearing of nematodes that emerged from spittlebugs until the adult stage was not accomplished, so the full morphological identification of these Mermithidae was not possible. Molecular analysis of nematodes showed the presence of two significantly different amplicon lengths of the ITS locus, one of about 950 bp and the other of about 780 bp. Interestingly, all samples collected in sites A and B share the same ITS locus length (950 bp), while the other samples from sites C, D, and E showed the shorter one (780 bp). According to the analyzed samples, the two species remained distinctly distributed without overlapping.

**Table 2:** *Philaenus spumarius* specimens parasitized by Mermithidae in Central Italy. The number of dissected spittlebugs, examined instars, and detected nematode specimens is listed according to the sampling site.

		***Philaenus spumarius***		**Mermithidae**			
**Site**	**Coordinates**	**No. of dissected specimens**	**Examined instar**	**No. of observed nematodes**	**No. of nematodes per spittlebug**	**Parasitization rate (%)**	**Species**
A^a^	43.938753N	411	n1, n2, n3, n4, n5, adult	94	1–4	22.87	1
	11.141433E						
B	43.811145N	516	n2, n3, n4, n5, adult	17	1	3.29	1
	11.031118E						
C^a^	43.732465N	322	n1, n2, n3, n4, n5, adult	16	1–2	4.97	2
	11.254696E						
D^a^	43.66854N	436	n1, n2, n3, n4, n5, adult	81	1–9	18.58	2
	11.152888E						
E^a^	43.799643N	322	n1, n2, n3, n4, n5, adult	17	1–5	5.28	2
	11.403262E						
F	43.761272N	402	n1, n2, n3, n4, n5, adult	0		-	-
	10.452208E						
G	42.737900N	400	n2, n3, n4, n5, adult	0		-	-
	11.05498E						
H	43.508942N	341	n1, n2, n3, n4, adult	0		-	-
	11.874063E						

a Sampling site where more than one mermithid was found in a single *P. spumarius* individual.

The sequencing of one randomly chosen ITS amplicon of each length showed that neither sequence had a sufficient identity percentage (%ID) to be assigned with certainty to a genus. Sequences of the 18S gene and the D2-D3 region of the 28S gene showed the same uncertainty in the identification of species.

The species with the longer ITS locus showed an 18S gene 98.8% ID with an unidentified Mermithidae Accession LC788413 (coverage 1717 bp) and no significant results for the D2-D3 locus. However, the closest identified genus was *Hexamermis* sp. LC661691 with 99.1% ID and 1638 bp coverage. This taxon was tentatively named “Species 1”.

The species with shorter ITS locus showed an 18S gene 98.9% ID with an unidentified Mermithidae isolate ATSA09b Accession OR614373 (coverage 1703 bp) and 93.7% ID with an unidentified Mermithidae isolate Nakatsugawa Accession LC788417 (coverage 764 bp). The closest identification genus was *Amphimermis* sp. A-2007 EF617372 (coverage 326 bp) with 96.9 %ID in the 28S gene fragment. This second taxon was named “Species 2”. Sequences were submitted to GenBank with accession numbers PQ811687-PQ811688 and PQ811689-PQ811690. A deeper characterization was attempted by constructing a phylogenetic tree that included both species; however, the result was biased by the uneven distribution of sequences present in GenBank and the gene fragment considered in the reconstruction. For the strong bias in the results, elaborations were not included in this work.

In most cases, a parasitized *P. spumarius* specimen hosted a single nematode, but multiple mermithids were occasionally counted in a single spittlebug. More specifically, for Species 1 in the sampling site A, 2–5 mermithids were observed in 15 of 94 parasitized *P. spumarius* specimens. For mermithid Species 2, superparasitization was recorded in sites C, D, and E, where 2–9 nematodes were counted in 59, 5, and 5 parasitized spittlebugs, respectively (Table 2).

The observed parasitization rate for Species 1 was 3.29–22.87% and for Species 2, 4.97–18.58%. Statistical analysis indicated that for Species 1, the parasitization rate recorded in site A was significantly higher than that observed in site B (χ^2^ = 83.18, df = 1, *p* < .000001). For Species 2, the parasitization rate in site D was significantly higher than that observed in sites C and E (χ^2^ = 60.29, df = 2, *p* < .000001).

## Discussion and Conclusion

The parasitization of *P. spumarius* by Mermithidae nematodes was observed for the first time in Central Italy, evidencing two distinct species of these nematodes infecting both nymphs and adults of the meadow spittlebug. To our knowledge, this work represents the first report of Mermithidae infecting *P. spumarius* in Europe.

The two nematode species reported in this study can be distinguished using a homology search in the GenBank database, attributing with a degree of caution Species 1 to the genus *Hexamermis* and Species 2 to the genus *Amphimermis*. However, future studies aimed at rearing adult Mermithidae from parasitized *P. spumarius* are needed to identify nematodes to the species level, since morphological traits essential for classical taxonomy are visible only in post-parasitic juveniles and adult mermithids.

In this study, nematodes were found infecting early-instar nymphs of *P. spumarius*. Based on these observations, it can be hypothesized that pre-parasitic juveniles attack spittlebug immature stages soon after they hatch from the egg. It might be possible that the infection occurs when pre-parasitic juveniles encounter early instar *P. spumarius* nymphs, which crawl on the ground in search of a suitable feeding site or feed on low plant rosettes (Cornara et al., 2018). Alternatively, mermithids may climb on plants and penetrate nymphs that are already feeding on herbaceous plants, since they are not very mobile. Although spittlebug nymphs are protected by a self-produced froth, this envelope may not provide effective defense against mermithids, as seen with attacks from entomopathogenic nematodes (Vicente-Díez et al., 2021; El-Khoury et al., 2024).

Likely, nematodes may enter *P. spumarius* nymphs by directly penetrating their thin integument. This mode of entry into the host has been documented for several Mermithidae (Nickle, 1972; Molloy and Jamnback, 1975; Mazza et al., 2017). Mermithid nematodes develop inside spittlebugs until the end of their preimaginal development. Indeed, newly emerged *P. spumarius* adults were observed carrying enlarged nematodes that probably were ready for molting into post-parasitic juveniles to be followed by emergence from the host. Since mermithids were not detected in adult spittlebugs collected in the second half of June (data not shown), it can be supposed that post-parasitic juveniles exited their hosts at the beginning of summer, before spittlebugs were ready to transition from herbaceous plants to shrubs and trees (Cornara et al., 2018).

Recorded parasitization rates for the two nematode species vary widely between sampling locations, with two peaks: 22.87% for Species 1 and 18.58% for Species 2. These upper levels appear to be consistent with previous studies on mermithid prevalence in insect host populations, where different parasitization rates varied with sampling sites, season, year of sampling, host sex, and instar (Choo et al., 1989; Baker and Xavier, 1998; Stubbins et al., 2017). Knowledge about abiotic and biotic factors influencing the presence, distribution, and infectivity of terrestrial Mermithidae is scarce. To date, soil characteristics and moisture, together with the availability and abundance of potential hosts, appear to be major drivers of Mermithidae occurrence and diversity (Hurpin and Robert, 1975; Senthil Kumar et al., 2018; Kaiser, 2020). In this study, the two putative nematode species seemed to be distributed in different areas of the Tuscany region without overlapping. This pattern is likely related more to specific environmental requirements of the single Mermithidae species than to the presence and abundance of *P. spumarius*. Furthermore, the existence of other hosts, which might be even preferred compared to *P. spumarius*, must be considered when discussing the observed distribution of the two mermithids found in this study.

In many cases, mermithid infection has epizootic traits, with more than 50% mortality in some insect host populations (e.g. Mongkolkiti et al., 1971; Nickle, 1981; Poinar and Linares, 1985; Toepfer et al., 2009). Therefore, the potential of Mermithidae as biological control agents has been explored in various studies, particularly for the management of pests like grasshoppers, beetles, and lepidopterans (Kaiser, 2020). Moreover, promising results were obtained using aquatic mermithids, like *Romanomermis* spp., for the control of several mosquito species in the genera *Anopheles*, *Aedes*, *Culex*, and *Mansonia* (Paily and Balaraman, 2000; Perez-Pacheco et al., 2015; Abagli et al., 2019). However, practical applications of Mermithidae are still infrequent because of the difficulty in taxonomic identification, mass rearing, and field release of these nematodes. All these issues are due to inadequate information about their biology and ecology (Petersen, 1985; Senthil Kumar et al., 2018).

Data collected in this work does not lead to consider the two nematode species found in *P. spumarius* as potential biological control agents: more in-depth research will certainly be necessary. Completing the taxonomic identification of these Mermithidae and understanding their ecological requirements could significantly improve the knowledge of their bio-ethological characteristics, elucidating their possible role in impacting spittlebug populations.
